# Characterization of Low-Cost Particulates Used as Energy Storage and Heat-Transfer Medium in Concentrated Solar Power Systems

**DOI:** 10.3390/ma15082946

**Published:** 2022-04-18

**Authors:** Rageh S. Saeed, Abdulelah Alswaiyd, Nader S. Saleh, Shaker Alaqel, Eldwin Djajadiwinata, Abdelrahman El-Leathy, Syed Noman Danish, Hany Al-Ansary, Sheldon Jeter, Zeyad Al-Suhaibani, Zeyad Almutairi

**Affiliations:** 1 Mechanical Engineering Department, King Saud University, P.O. Box 800, Riyadh 11421, Saudi Arabia; a.alswaiyd@gmail.com (A.A.); nader.alabsi@gmail.com (N.S.S.); shaker_alaqel@windowslive.com (S.A.); eldwin_dj@yahoo.com (E.D.); aelleathy@ksu.edu.sa (A.E.-L.); hansary@ksu.edu.sa (H.A.-A.); zeyads@ksu.edu.sa (Z.A.-S.); zaalmutairi@ksu.edu.sa (Z.A.); 2 K.A.CARE Energy Research and Innovation Center at Riyadh, Riyadh 11451, Saudi Arabia; 3 Mechanical Power Engineering Department, Faculty of Engineering, El-Mataria, Helwan University, Cairo 11718, Egypt; 4 Sustainable Energy Technologies Center, King Saud University, P.O. Box 800, Riyadh 11421, Saudi Arabia; 5 Georgia Institute of Technology, School of Mechanical Engineering, 771 Ferst Drive, Atlanta, GA 30332, USA; sheldon.jeter@me.gatech.edu

**Keywords:** concentrated solar power, particulates, thermal energy storage, heat transfer medium, characterization

## Abstract

Utilizing solid particles as a heat-transfer medium in concentrated solar power applications has gained growing attention lately. Unlike molten salts, solid particles offer many benefits, which include: high operating temperatures (greater than 1000 °C), a lack of freezing issues and corrosivity, abundant availability, high thermal energy storage capacity, a low cost, and applicability in direct irradiation. Comprehensive knowledge of thermophysical and optical properties of solid particles is essential to ensure an effective harnessing of solar energy. The most important considerations when selecting solid particles include: thermophysical and optical properties, thermal resistance, crack resistance, satisfactory health and safety risks, availability, and low cost. It is also imperative to consider optical and thermophysical characteristics that might change from what they were “as received” after cyclic heating for a long period. Therefore, the knowledge of thermal performance of particulate materials becomes significant before using them as a heat-transfer medium. In this study, some particulate materials were chosen to study their feasibilities as heat-transfer and storage media for a particle-based central receiver tower system. These particulate materials included white sand, red sand, ilmenite, and Carbobead CP. The candidate particulate materials were heated at high temperatures for 6 h and then cooled to room temperature. After that, cyclic heating was performed on the particulate materials for 500 h at 1200 °C. The optical properties were represented by weighted solar absorptance, and the thermophysical properties of the particulates were measured “as received” and after cyclic heating (aging). EDX and XRD were conducted to quantify the chemical composition and interpret the changes in appearance associated with the particulate materials after cyclic heating. The results showed a considerable agglomeration in all particulates except for white sand in the 6 h heating test, and high agglomeration in the ilmenite. A slight decrease in the optical properties in the white sand and Carbobead CP was found after the aging test. The specific heat was decreased for red and white sand. The EDX and XRD results for white sand and Carbobead CP showed chemical stability, indicating high durability and reliability.

## 1. Introduction

The technology of the particle-to-working fluid heat exchanger (PWFHX) is closely related to central receiver solar technology. The idea of a central receiver system based on gas and other power-generation cycles has gained considerable interest during the past two decades, due to its capability of achieving very high temperatures through intense heat-flux concentration in a relatively small area. Various gas-cycle concepts have been proposed and tested, and most involve the direct heating of compressed air or other gases [1,2,3]. However, one of the major challenges of these systems is the successful incorporation of thermal energy storage (TES), since the effectiveness of using TES with air or gas is relatively poor.

There have been several TES solutions proposed over the past three decades. Ho [4] reviewed and summarized the recent advances in solid-particle-based central receivers. One of the most widely accepted TES solutions is the use of molten salts [5,6]. Currently, the use of molten salts for thermal energy storage is limited to temperatures generally less than 600 °C due to technical restrictions. Another solution is the use of solid blocks to store energy during the day. This idea has been demonstrated with concrete blocks [7,8], but the temperatures are generally limited to less than 500 °C due to concrete’s properties, making this concept unsuitable for high-temperature applications. Furthermore, since solid blocks store sensible heat, their temperature profile during the discharging process causes a gradual decline in cycle efficiency. Yet another solution is to use sand as a storage medium [9]. This concept was developed to work in conjunction with an air receiver. The sand is heated in an air–sand heat exchanger to a very high temperature. The sand then flows to a hot storage tank, and then to a fluidized bed cooler, where its heat is used to generate steam that feeds a steam power cycle. The colder sand returns either to the air–sand heat exchanger or is stored in a cold storage tank. This technology resolves the temperature limit issues faced in the solid-block concept. However, the main issue of an unfavorable temperature profile during discharging still persists.

TES in solid particles has been studied by a number of research groups. The research originated at Sandia National Laboratories (SNL) in the early 1980s examined the use of solid carriers as both a storage medium and working fluid for high temperatures [10,11,12,13,14,15,16,17]. The emphasis was on particle material selection and receiver design, along with the optical characterization of master beads and other particle materials. TES materials were also investigated in the past for varying applications. Zunft et al. [18] quantified the heat losses from a subsystem TES, which consisted of rectangular storage composed of four parallel chambers filled with the ceramic storage material studied in the industrial regenerative thermal oxidizer. The temperature at full load reached 630 °C, and the total heat losses in a 24 h period amount to 930 kWh. An experimental packed-bed TES designed by CIEMAT-PSA [19] was composed of an insulated stainless-steel vessel filled with 0.1 m^3^ alumina spheres with a 9 mm diameter to investigate the specific costs of the storage subsystem under 20 EUR/kW_th_. An air inlet temperature of 570 °C and different air mass flow rates have been investigated.

Tescari et al. [20] evaluated the mechanical properties of structured reactors/heat exchangers in high-temperature heat storage via the cobalt oxide cyclic-redox scheme. Two different structures of honeycomb and perforated block and two different compositions were evaluated. During thermal cycling in the range of 800–1000 °C, different loads were applied to the samples while monitoring their length variations. Calderón et al. [21] reviewed solid-particle materials to be used as both a heat-transfer fluid and a medium for thermal energy storage. The parameters and properties of solid particles were described from a materials science point of view by illustrating their function and their connection to the performance of the power plant and its durability. The interactions between solid particles and major system components were further discussed in this review. Furio et al. [22] evaluated two coating methods for silica sand to improve the optical properties. They reported a 140% increase in optical properties. Specific heat capacity and energy density for different particulate materials were studied by Kang et al. [23]. These particulate materials included silica sand, quartz sand, cristobalite, alumina, sintered bauxite, silicon carbide (SiC), and olivine sand. The results showed significant differences between the various materials, and that the olivine sand had the most favorable characteristics, followed by alumina and cristobalite. Proppants have good optical and mechanical properties to be used as heat transfer medium in concentrated solar power (CSP) systems [24]. They are characterized by a high solar weighted absorptance higher than 90% “as received”, and they are noncorrosive materials. Proppants are available in large quantities; therefore, their cost is relatively low. However, fresh proppants are chemically unstable in oxidizing environments, and their solar weighted absorptance decreases when heated in air at 700 °C for many hours. Siegel et al. [24] measured the radiative properties, solar weighted absorptance, and thermal emittance for different manufactured types of proppants, including CARBOHSP, CARBOACCUCAS, CARBOPROP, and Norton Masterbeads. The measurements of these materials were conducted in two different states, the “as received” state and after heating in the air between 700 °C and 1000 °C. The results showed a significant reduction in solar weighted absorptance in the “as received” particles over time and temperature. The effects of aging on the optical and thermal properties were studied for silicon carbide (SiC), silica sand (SiO_2_), and hematite (Fe_2_O_3_) by Palacios et al. [25]. The aging tests were conducted for 500 h at 750 °C and 900 °C. The results showed that the weighted solar absorptance for black SiC and iron oxide increased and decreased for SiO_2_ with time. The highest absorptance and specific heat capacity values were recorded for the black SiC after aging at 900 °C for 500 h. Nie et al. [26] measured the thermophysical and optical properties of nine different solid-particle samples to be used in a circulating solid-particle receiver. Those were alumina, silicon carbide, quartz sand, desert sand, river sand, yellow ceramist sand, grey ceramist sand, bauxite–cordierite ceramics, and black copper slag. Silicon carbide, grey ceramist sand, and bauxite–cordierite ceramics were found to be appropriate materials after the consideration of many factors. The most important factor was the high solar weighted absorptivity, even after heat treatment. Thermophysical, optical, and mechanical properties were measured for several particulate material samples extracted from different locations in the United Arab Emirates by Diago et al. [27]. The results showed that the particulate samples appeared stable at temperatures ranging from 650 °C to 1000 °C. Solar absorptance decreased due to the particle phase change in which calcium carbonate was transformed into calcium oxide after heating to high temperatures. Aging tests for 500 h at 900 °C and thermal cyclic for 1500 cycles between 300 °C and 900 °C were conducted on silicon carbide (SiC) and CarboHSP^®^ 30/60 by Calderon et al. [28]. Tests to determine solar absorptance, chemical composition, physical properties, and thermal characteristics, as well as morphologic analysis of the samples before and after thermal treatments were performed. The characterization results showed that SiC was more affected in its durability by thermal cycling than by constant-temperature aging treatment, while CarboHSP^®^ was affected by temperature aging rather than thermal cycling. SiC reacted with oxygen to form SiO_2_ on the surface, with a positive effect on its solar absorptance. Nevertheless, with thermal cycles, the SiC particle surface became damaged, and the reaction continued with the additional newly exposed surface. Meanwhile, CarboHSP^®^ reduced its solar absorptance with the high temperatures only due to changes in its surface chemical composition. However, thermal cycling showed no negative effect on the properties of CarboHSP^®^. Three different granular materials were characterized and tested by Díaz-Heras et al. [29] for use as heat-transfer fluids and storage materials in CSP systems: sand, carbo Accurist ID50, and silicon carbide. Thermal aging at 900 °C for 500 h was conducted. The samples were also fluidized for 250 h at twice the minimum fluidization velocity (at ambient temperature). The properties of the particles before and after fluidization and before and after heating to a high temperature (900 °C) were investigated. The results showed that there was no significant change in their chemical compositions, and their specific heats and absorptivities did not vary notably after the tests. The main attractive property of SiC and Carbo is their very high absorptivity, which is around or over 0.9, while for the sand it is around 0.4. However, the abundance of sand makes it cheaper than SiC and Carbo by 4–5 times. Furthermore, sand’s lower density permits its particles to be transported/fluidized with lower pumping costs.

Silica sand or similar mineral particulates can be an inexpensive, stable, and environmentally benign TES medium in CSP or other high-temperature systems. Silica sand or other similar suitable particulates are stable well above 1000 °C, while common nitrate salts begin to decompose at 600 °C or lower, and typical organic fluids cannot be used much above 400 °C. Heat transfer to and from a static bulk solid usually results in considerable temperature degradation. However, the development of an efficient exchanger that handles flowing sand in general counterflow to the collection or working fluid will greatly reduce this degradation. Indeed, our recently completed project sponsored by the US Department of Energy showed that sand can be used as both a heat-collection and TES medium in a CSP system by heating the sand directly in a particle heating receiver (PHR) system [29].

Preparations are underway to implement the improved modified concept in a pre-commercial integrated PHR–gas turbine system with a 1.3 MW_e_ rated capacity and six hours of thermal energy storage, making the incident thermal power of the PHR on the order of 7 MW_th_ with an operating temperature of 1200 °C [30]. Such temperature durability and reliability of HTMs is very important. Three natural and low-cost materials were studied; namely white sand, red sand, ilmenite, and one engineered particulate material called Carbobead CP. This work was a continuation study of a TES system that was built at King Saud University, Riyadh, Saudi Arabia [31,32,33,34,35,36,37,38,39,40,41,42,43,44,45]. With this work, we have extended our ongoing efforts in experimentation and modeling various options to enhance the heat transfer to or from sand or other particulates. The effect of aging on the particulate materials, optical properties, and thermophysical properties were studied. The results of this study could lead to a major reduction in the critical costs of a promising TES concept.

## 2. Selection of Particulate Materials

**Figure 1:**
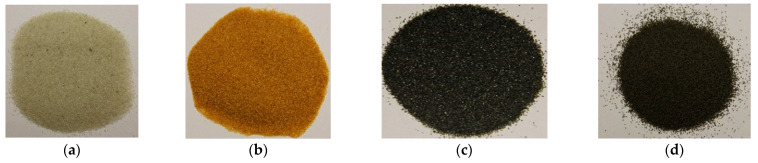
Candidate particulate materials: (**a**) Riyadh white sand; (**b**) Riyadh red sand; (**c**) ilmenite; (**d**) Carbobead CP.

The key enabling technology in this study was the particle heating receiver (PHR), since it has potentially very high efficiency, can achieve higher temperatures than any other competing technology, and can be built cost-effectively. An integral part of the PHR subsystem is the particulate material that is fed through it to be heated by concentrated sunlight. This material needs to strike the right balance between: (a) cost, (b) optical properties, and (c) availability in the local market. Three different natural and local particulate materials (Riyadh region): white sand (WS), red sand (RS), and ilmenite; and one engineered particulate material: Carbobead CP, were considered. Figure 1 shows samples of the four materials.

All samples were cleaned and dried to ensure that: (a) the samples were free from contamination, and (b) the particle sizes were within the recommended size range in the real operation, such that the important system components were not clogged with large particles. The particulates then were sifted using a vibrator sieve machine (ETA Vibro Screen) available at King Saud University, while the Carbobead CP was supplied with a standard and uniform particle size. Two meshes sizes were used in the sifting process (40 and 70), such that the particles were passed from 40 mesh (which only allowed particles smaller than 425 microns to pass) and stopped at 70 mesh (which did not allow particles larger than 212 microns to pass). After sifting with the 40/70-mesh sieve, the particle sizes were 210–400 μm. The engineered ceramic particles were from CARBO Industrial Technologies(Houston, TX 77041, USA). Carbobeads are engineered particles with a high shape uniformity (sphericity of 0.9), whereas the other particulate materials’ grains typically had an irregular shape. Table 1 qualitatively describes the merits of each of the materials.

In order to achieve this study’s objectives, the experimental work was conducted on selected particulate materials to be used as heat-transfer media in the obstructed flow PHR. The candidate particulates were tested “as received” and after cyclic heating for a certain duration of time. Details on how the experiments were done are presented in the following sections.

## 3. Methodology

### 3.1. Sustainability Test at High Temperatures (Aging Test)

In this part, aging tests were performed on the candidate particulate materials in order to simulate the cyclic heating that particulates encounter under the operating conditions in particle-based CSP systems. These tests aimed to investigate the aging effect on the optical, mechanical, and thermophysical properties. The tests were conducted at 800, 1000, and 1200 °C. These temperatures were the designed operating temperatures in such systems. Each particulate material type was divided into four samples, and the first sample was kept untreated and used as a reference. The other three samples were put in the ceramic crucibles and heated in the Muffel electric furnace (Hindustan Thermostatics, Haryana, India) to 800, 1000, and 1200 °C in an air environment for 6 h at a heating rate of 10 °C/minute, then left to cool down to room temperature. The agglomeration behaviors of the particulate materials was the first sign used to select which particulate would be subjected to cyclic heating later. The samples of each material, which were initially heated to 1200 °C and did not show any sign of agglomeration, were reheated to 1200 °C for 8 h and then left to cool down to room temperature. The last process of heating to 1200 °C for 8 h and cooling down to room temperature was continued until 500 h of cyclic loading was achieved. These tests simulated an extreme case of a “6 h” storage system.

### 3.2. Elemental Analysis

It is important to investigate the effect of cyclic heating on changes in optical appearance and color problems, adhesiveness, and bonding by verifying the chemical composition of the particulate materials. Identification of the cause of the agglomeration behavior was conducted by confirming whether the agglomerated masses that were found were impurities in the sand or something else that was formed during the cyclic heating. Energy-dispersive X-ray analysis (Jeol Limited, Akishima, Tokyo, EDX), together with a scanning electron microscope (Jeol Limited, Akishima, Tokyo, SEM) both by Jeol Limited (Akishima, Tokyo), were used to identify the elemental composition of the particulate materials. Qualitative and quantitative analyses were conducted to identify the types of elements that were present, as well as the percentage of each element’s concentration within the sample. Furthermore, an X-ray diffractogram (Billerica, Massachusetts, MA, USA, XRD) analysis was done using a Bruker D8 Discover multipurpose X-ray diffractometer (Billerica, Massachusetts, MA, USA) with CuKα source radiation to determine the crystallographic structure of the materials, as well as to determine the physical mechanisms responsible for the optical properties (color change) of the particles after exposure to heating. Bruker EVA^TM^ (Billerica, Massachusetts, MA, USA) was used for the matching and analyzing and interpretation of the XRD results. These tests and analyses were conducted on candidate particulate materials “as received,” and on each heated sample (to 800 °C, 1000 °C, 1200 °C—6 h, and 1200 °C—500 h). The XRD patterns were obtained at room temperature.

### 3.3. Optical Properties

**Figure 2:**
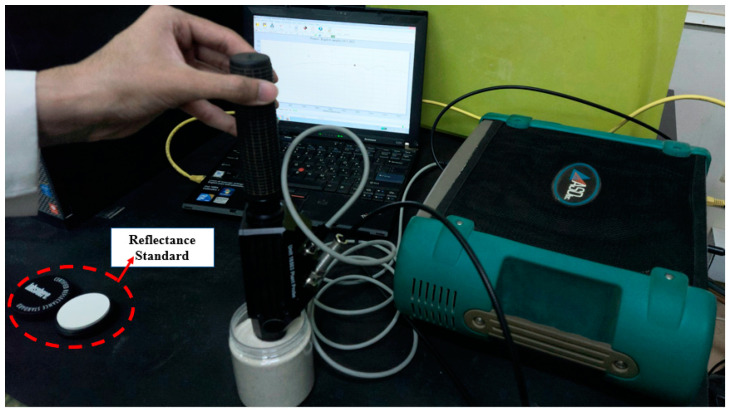
Reflectivity measurement of particulate materials using ASD FieldSpe3 Portable Spectroradiometer device.

The reflectivities of the candidate materials were measured using the ASD FieldSpe3 Portable Spectroradiometer device (Malvern Panalytical company, Cambridge, United Kingdom) shown in Figure 2 in the 350–2500 nm wavelength range. The instrument was calibrated with a certified reflectance standard provided by the Labsphere company. The tests were performed in a dark area to prevent the effects of reflection or diffusion of ambient light on the measurements. A sufficient thickness of each tested particulate material was placed on the sample stand to make sure that the stand was completely covered with particulates and light reflected to the sensor was only from the sample and not from the base of the stand. This procedure was performed to make sure that the light transmission was equal to zero. All of the reflectivity measurements for each sample were repeated following this protocol seven times. All of the particulate reflectance measurements were conducted at ambient temperature. Equation (1) was used to calculate the absorbance:

(1) $$R_{\lambda }+T_{\lambda }+A_{\lambda }=1$$

where *R*, *T*, and *A* indicate the reflectance, transmittance, and absorptance, respectively; while *λ* indicates the wavelength at which the measurement was performed. Considering a transmittance equal to zero, the absorptance was found using Equation (2):

(2) $$A_{\lambda }=1-R_{\lambda }$$

To find the weighted solar absorptance of the candidate particulate materials, the absorptance values were weighted with respect to the values of the solar spectrum according to ASTM G173-03 and the air mass of AM1.5, as shown in Equation (3) [25]:

(3) $$A_{\mathrm{weighted}}=\mathrm{AM}1.5\;\left(W/{\;m}^{2}\mathrm{nm}\right)\cdot \;A_{\mathrm{measured}}$$

Finally, a trapezoidal integration was executed under the curve to calculate the total absorptance of the material:

(4) $$\mathrm{Total}\;\mathrm{Absorptance}=\overset{N}{\underset{i=1}{\sum }}\frac{\frac{A_{1}+A_{i+1}}{2}}{N-1}$$

where $A_{i}$ is the first low range value, $A_{i+1}$ is the higher range value, and *N* is the number of values.

### 3.4. Thermophysical Properties

#### 3.4.1. Bulk Density

The bulk density (loose) of the candidate particulate materials for each test was measured. The mass of the solid particles was measured using a high-accuracy KERN PLJ 4000-2M lab scale, whereas the volume of the solid particles was measured using a high-accuracy graduated PYREX beaker. The bulk density was obtained using the following procedure: (1) the particles were poured into the beaker and the mass was measured; (2) the volume occupied by the particles as indicated by the graduated beaker was noted; and (3) the bulk density was calculated by dividing the total mass by the total volume. The measurement of the particle density was done using Archimedes’ principle, in which distilled water was used to fill the void fraction between the solid particles. The density of the distilled water was measured using the KD2 PRO device at different temperatures starting from ambient temperature to 70 °C. In order to determine the exact particle volume, the distilled water was put in the beaker and then weighed. The water volume was calculated by dividing the water mass by the measured density. The particle sample then was poured freely into the distilled water to ensure that the particle was surrounded by water on all sides and to avoid forming air bubbles between the particles and the distilled water. The particle mass was calculated by subtracting the measured water mass from the total mass. In addition, the particle volume was determined by subtracting the calculated water volume from the total volume. The particle density was finally calculated by dividing the particle mass by the particle volume. All of the measurements were taken in atmospheric air and at ambient temperature.

#### 3.4.2. Specific Heat

The specific heat capacity (c_p_) is an important property to be considered in the selection of particulate materials that are going to be used as a medium for thermal energy storage in particle-based CSP systems. The higher the specific heat capacity, the higher the heat storage capacity that can be achieved. The specific heat was measured for only the white sand and red sand samples “as received”, and after cyclic heating at 1200 °C as a function of temperature using the differential scanning calorimetry (DSC) technique at Sandia National Laboratory (SNL). The measurements were conducted from 35 °C to 1200 °C under an argon atmosphere with a flow of 80 mL/min and at a heating rate of 10 (K/min) in a NETZSCH STA 409C/CD. A platinum–rhodium crucible (DSC/TG pan Pt-Rh) was used to hold the samples in these tests. The amount of each sample used was around 20 mg. All of the specific heat measurements were calibrated using a crystal of sapphire provided by NETZSCH. The measurements were extremely sensitive to experimental factors (e.g., crucible size, shape, mass, placement within the furnace, matching with the reference crucible, and sample characteristics such as distribution within the crucible and sample mass). In addition, the matching of each sample’s raw DSC signal to that of the sapphire standard was taken into account during the calibration and c_p_ measurements of the samples.

## 4. Results and Discussion

### 4.1. Effects on Optical and Thermophysical Properties of Particulates

#### 4.1.1. Particle Agglomeration

Photographic images of the candidate particulate materials “as received” and after heating are shown in Table 2. In the beginning, three samples from each natural particulate material—Riyadh white sand, Riyadh red sand, and ilmenite—were heated to 800, 1000, and 1200 °C for 6 h and then cooled to ambient temperature. The results did not show any signs of agglomeration in all the white sand samples. In the case of the red sand, the sample heated to 800 °C did not show any signs of agglomeration, while a very weak adhesion between the particles was observed in the red sand after heating it to 1000 °C. A weak agglomeration began to form in the sample that was heated to 1200 °C. Generally, the agglomeration at 1200 °C was not significant, and it was easy to separate the agglomerated particles. The color of the sand was found to have become darker after heating. This was probably due to the removal of moisture and other impurities from the sand. The ilmenite particles began to show some signs of agglomeration at 800 °C, but when the ilmenite was heated to 1000 °C, large lumps were created. Lump formation increased at 1200 °C and was very hard to break. This agglomeration became harder at 1200 °C. Based on the aforementioned observations, the ilmenite was excluded from the next heating stage and replaced with the Carbobead CP sample for evaluation.

The samples of white sand and red sand, which were heated to 1200 °C initially, were reheated to 1200 °C for 8 h and then cooled down to room temperature. Three samples of the Carbobead CP were tested at 1200 °C for different periods: one for 4 h, one for 32 h, and one joined to the white sand and red sand in the cyclic heating test. This cycle was repeated until reaching 500 h of heating to observe the aging effect on each material. The results also showed that the agglomeration did not appear in the white sand sample, while a slight agglomeration occurred in the Carbobead CP sample. However, the agglomerated masses were very easy to break. A hard agglomeration appeared in the red sand sample, and formed a large lump.

#### 4.1.2. Particle Color Change

Table 2 shows the gradual change in the appearance of the tested samples with temperature. The results showed a bit of change in the color of the white sand samples, which became more whitish. In the case of the red sand, the appearance of all the samples slightly changed and became darker after heating at 800, 1000 and 1200 °C for 6 h. After the aging process at 1200 °C for 500 h, the results showed that the white sand turned more whitish. In the case of the red sand, a significant degradation in its appearance occurred, in which the color changed entirely to white. The Carbobead CP exhibited a small color change compared to the original color, experiencing a slight darkening of its color to a tan or orange particle color. Changing the color of the particulate materials and agglomeration after cyclic heating was interpreted using EDX and XRD analyses. In general, the particulate samples appeared to be stable at temperatures ranging from 650 °C to 1000 °C. However, we cannot say that these particulate samples were not affected by heating, because they were heated for only 6 h and were not exposed to the cyclic heating at 800 and 1000 °C.

**Figure 3:**
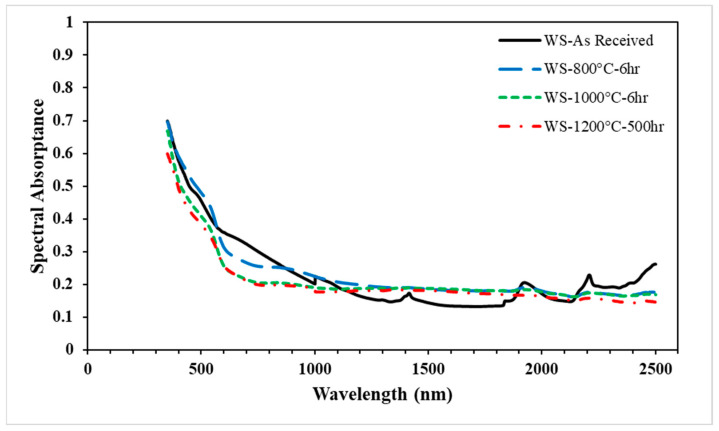
Spectral absorptance of the white sand samples (“as received” and after the aging test) over the wavelength.

The spectral absorptivity of white sand over a wavelength range of 350–2500 nm is represented in Figure 3. The results show a variation of spectral absorptance for white sand “as received” and after each heat treatment. In all the samples, the spectral absorptance deviated slightly from the “as received” condition following heating at 1200 °C for up to 500 h. Generally, the spectral absorptance decreased with increasing wavelengths until reaching 900 nm, and then became almost constant. The results also showed the spectral absorptance of the aged sample, which became more stable within the whole wavelength. The results showed that the white sand sample heated to 800 °C did not show any change from the values “as received”. A slight drop in the solar absorptance appeared after heating to 1000 °C for 6 h, while a slight drop after cyclic heating at 1200 °C for 500 h was noted compared to the sample heated at 1000 °C for 6 h. The solar weighted measurements of the white sand, red sand, and ilmenite particulates are presented in Table 3.

**Figure 4:**
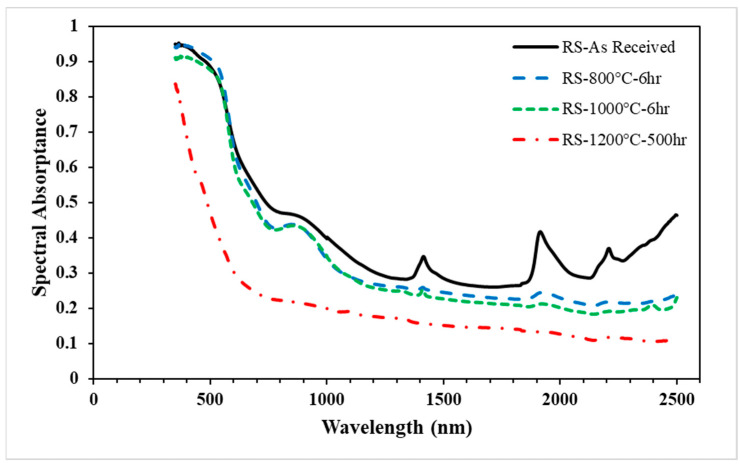
Spectral absorptance of the red sand samples (“as received” and after the aging test) over the wavelength.

The spectral absorptivity of the red sand over a wavelength range of 350–2500 nm is presented in Figure 4. The results show that the spectral absorptance of the red sand continued to decrease as the heating exposure time increased. Unlike the white sand, the decrease in spectral absorptance was sharp at wavelengths less than 1200 nm, and did not reach a steady value even after the 500 h test. The peaks that appeared in the “as received” sample may be attributed to the impurities in the red sand that disappeared after heating at high temperatures. Table 3 shows that the solar absorptance results for the red sand deteriorated significantly over time and temperature. The decrease in the absorptance was small at 800 and 1000 °C, but a strong deterioration in solar absorptance was observed along with the color change after the cyclic heating test at 1200 °C for 500 h. The red sand lost 37% of its solar weighted absorptance from its value “as received” after cyclic heating at 1200 °C for 500 h.

**Figure 5:**
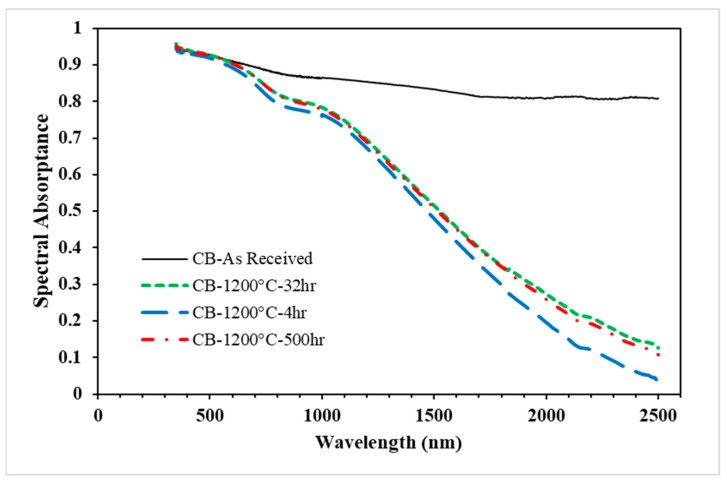
Spectral absorptance of the white sand samples (“as received” and after aging test) over the wavelength.

Figure 5 shows the variations in spectral absorptance of the Carbobead CP over a wavelength range of 350–2500 nm and under heat treatments at 1200 °C for different periods. The results show that the spectral absorptivity of the particles decreased as the wavelength increased, and does not reach a steady value within the 500 h test limit. The Carbobead CP that was heated for 4 h at 1200 °C still almost simulated the solar absorptivity of the “as received” particulate due to the short time of heating to which the Carbobead CP was exposed. The results showed an increase in the values of spectral solar absorptance with an increase in the heating exposure time. However, the change in optical properties with exposure time was considerably slower between 4 h of heating operation and the samples exposed to cyclic heating for 32 h and 500 h. The change in solar weighted absorptance for all three samples is shown in Table 3. The solar weighted absorptance dropped only 4% from the “as received” value after being exposed to cyclic heating at 1200 °C for 500 h.

### 4.2. Elemental Analysis

In order to determine the physical mechanism responsible for the observed change in color and in the optical properties of the particles that were subjected to cyclic heating, the candidate particulate materials were examined using EDX and XRD.

**Figure 6:**
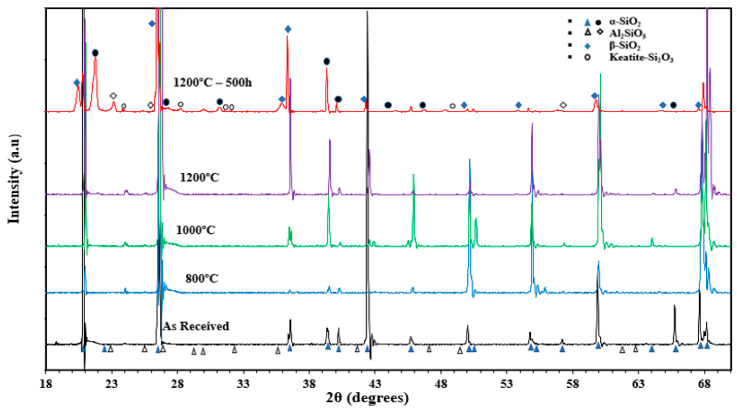
XRD data for white sand in the “as received” condition, following heating in air at different temperatures, and after cyclic heating at 1200 °C for 500 h.

The elemental composition of the white sand is tabulated in Table 4, while the XRD results are presented in Figure 6. The results show the major contents of the samples were silicon (Si) and oxygen (O_2_), with a small percentage of other elements such as aluminum. The EDX results show a decrease in the O_2_ weight percentage and an increase in the silicon weight percentage with temperature. The higher percentages of Si and O_2_ refer to the greater existence of silica SiO_2_ in the white sand sample and a very small percentage of Al_2_O_3_. The XRD analysis showed that all the peaks for all the samples represented SiO_2_. The changes in the intensity of the peaks were due to the exposure of the samples to high temperatures. It was clear that the peaks matched for the samples “as received” and the samples subjected to heat-treatment tests for 6 h, which indicated that the structural changes did not take place as a result of heating for 6 h. However, when the sample was exposed to cyclic heating for 500 h, structural changes took place. The results also show that the peaks of the samples “as received” and the samples subjected to heat treatment tests for 6 h were assigned to α-SiO_2_ as the major phase and aluminum silicate (Al_2_O_3_·SiO_2_) as the minor phases; while the β-SiO_2_ beside α-SiO_2_ was the major phase in the aged sample, and keatite (SiO_2_) and aluminum silicate (Al_2_O_3_·SiO_2_) were the minor phases.

The EDX results for the red sand (“as received” and after each heating test) are summarized in Table 5. The EDX results show that the red sand (as received) contained more elements than the white sand, indicating the presence of more impurities. Since the red sand had a minor component of ferrous oxide, it may have increased the chances for agglomeration at high temperatures. It was evident that the lesser the unwanted impurities in the silica, the lower the agglomeration would be. Like those for the white sand, the EDX results for the red sand showed a decrease in the O_2_ weight percentage with temperature. Table 5 also shows the presence of Fe in the red sand, whereas there was no Fe content in the white sand. The Fe disappeared after exposing the sample to cyclic heating at 1200 °C for 500 h. This meant that the change in color from red to white may have been due to evaporation of the Fe element in the sample, since Fe is what gave the red color to the sample of red sand. It is also possible that the color change upon heating could have resulted from a decrease in the density of oxygen and the corresponding change in the oxidation state of the metal ions in one or more of the red sand constituents that was not accompanied by a phase change. According to the EDX analysis shown in Table 5, the elements that disappeared in the aged sample were Fe, Ca, Mg, C, and K. On the other hand, only components of silica appeared in the sample after aging. A relevant qualitative observation was that the aged samples of white sand and red sand converted completely to pure silica.

**Figure 7:**
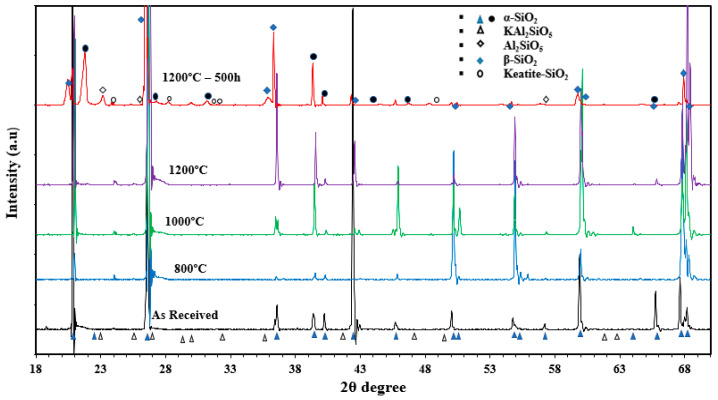
XRD data for red sand in the “as received” condition, following heating in air at different temperatures, and after cyclic heating at 1200 °C for 500 h.

The XRD data for red sand “as received”, as well as for the samples that were exposed to heat treatment, are shown in Figure 7. The XRD results obtained for the samples that were not exposed to cyclic heating showed a clear matching in the peaks, with a small difference in the intensities. This peak intensity may be attributed to the temperature that was applied when heating. All the peaks of the red sand samples matched with the silica pattern, which proved that all the samples represented a strong existence of SiO_2_. A few peaks were also suppressed in the sample that was exposed to cyclic heating at 1200 °C for 500 h. This might have been because the sand initially had some impurities that were removed during the cyclic heating process. Like those of the white sand, the peaks of the samples “as received” and the samples subjected to a heat-treatment test for 6 h were assigned to α-SiO_2_ as the major phase and potassium aluminum silicate (KAl_2_O_3_·SiO_2_) as the minor phases; while the β-SiO_2_ was the major phase in the aged sample, and α-SiO_2_ keatite (SiO_2_) and aluminum silicate (Al_2_O_3_·SiO_2_) were the minor phases. It was difficult to observe the phases associated with iron oxide, either Fe_3_O_4_ or Fe_2_O_3,_ or even Fe_2_TiO_5_, in samples that were not subjected to cyclic heating. This may have been due to the existence of these materials below the detection limit of the XRD.

**Figure 8:**
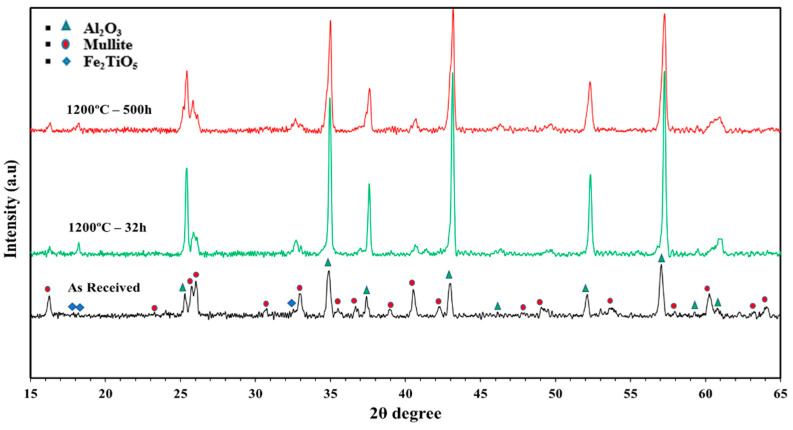
XRD data for CARBOBEAD CP in the “as received” condition and following heating in air at 1200 °C for different periods. Symbols correspond to the following phases.

The EDX and XRD analyses of the Carbobead CP samples “as received” and after cyclic heating at 1200 °C for 32 h and 500 h are summarized in Table 6 and presented in Figure 8, respectively. Unlike the white sand and red sand, the O_2_ weight percentage increased in all of the elemental weight percentages with temperature. The EDX data show a strong existence of Al in all the samples that increased by 20% from the “as received” sample. Conversely, the Si and Fe contents decreased with cyclic heating time. Only O, Al, Si, and Fe elements were detected in the sample after aging. The higher percentage of Fe in the “as received” sample gave it the dark color, thereby giving it a higher weighted solar absorbance. The EDX results show that the Si was decreased after heating to 1200 °C for 4 h, and then increased again upon further heating to 1200 °C for 32 h and 500 h.

Since EDX is an analytical technique that uses an electron beam that could penetrate from 1 to 2 micrometers, it is safe to say that the changes in the elemental weighted percentages were on the surface due to the diffusion of elements at higher temperatures. However, these results could not be interpreted for the bulk of the material.

The XRD results show that the peaks of the Carbobead CP samples (“as received” and the samples subjected to heat-treatment tests for 32 h and 500 h) were assigned to Al_2_O_3_ as the major phase and mullite and Fe_2_TiO_5_ as the minor phases. Although there was a high content percentage of the Fe in the samples (11% of the total CP mass “as received”), the phases associated with Fe_3_O_4_ and Fe_2_O_3_ were not observed in the XRD results. That did not mean that there was no change in the phases associated with iron oxide, but that the iron was most likely contained within other structures, such as Fe_2_TiO_5_. The slight change in color after heating may be attributed to an increase in the density of oxygen and a corresponding change in the iron oxide state.

### 4.3. Specific Heat

**Figure 9:**
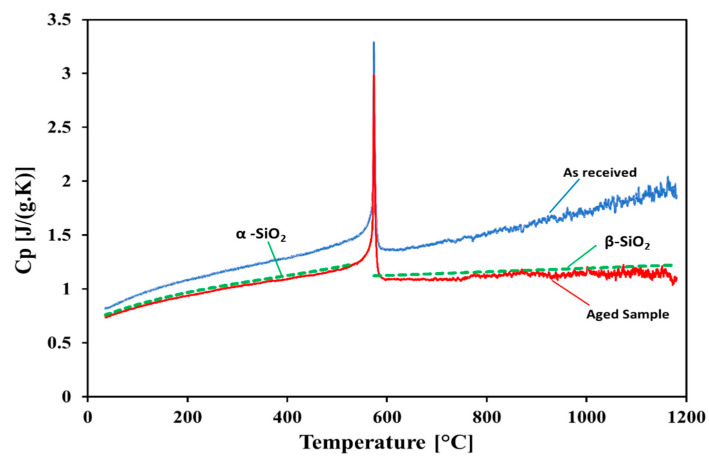
Specific heat measurements of the white sand sample.

**Figure 10:**
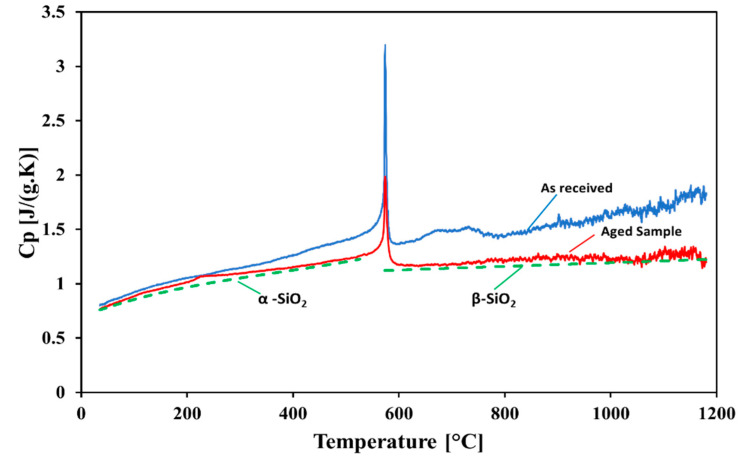
Specific heat measurements of the red sand sample.

The specific heats (c_p_) of the white sand and red sand samples (“as received” and after the cyclic heating test at 1200 °C for 500 h) are presented as a function of temperature in Figure 9 and Figure 10, respectively. The behavior of the cp was the same for the samples—it increased with the increase in temperature. The measured values of the c_p_ were higher for the “as received” samples in comparison with those of the aged samples. In the case of the “as received” samples, the specific heat of the red sand was lower than that of the white sand. For the red sand, it was obvious that the c_p_ values did not show any change below 200 °C after exposure to the cyclic heating at 1200 °C for 500 h. For the aged samples, the c_p_ values for the white sand were lower than that of the aged sample of red sand. After a phase change (<600 °C), the c_p_ of the aged sample of white sand became almost constant. The sharp pile at 573 °C identified the inversion of quartz as it transitioned from α-quartz to β-quartz due to the high SiO_2_ content in these samples [47]. The results show that the c_p_ values of the “as received” samples dropped after aging, and matched the c_p_ of the pure silica given in [48]. These differences may be attributed to the impurity contents or inhomogeneous composition of the phases in the particles. Hence, even after the aging treatment and proof via the EDX and XRD analyses that the white sand and red sand became almost all silica, we cannot regard them as pure SiO_2_ with a homogeneous quartz phase. 

### 4.4. Density

**Figure 11:**
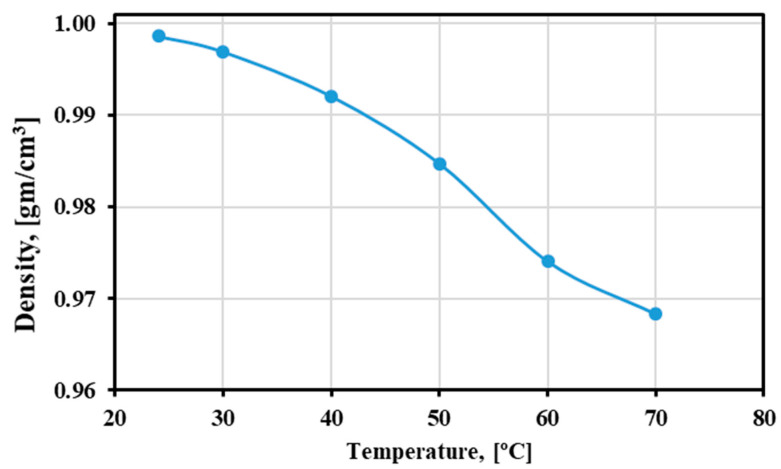
Density measurements of the distilled water with temperature.

The theoretical density results for the distilled water are plotted in Figure 11. The bulk density and particle density are summarized in Table 7. The results show an increase in both the densities. The highest increase was observed in the red sand after cyclic heating at 1200 °C for 500 h. The density of the CP decreased after 500 h from what it was after 32 h of cyclic heating.

## 5. Conclusions

Four different types of particulate materials were studied as a part of selecting suitable particulates to be used in particle-based CSP systems. These particulate materials were fracking Riyadh white sand, Riyadh red sand, ilmenite, and one engineered particulate material called Carbobead CP. Four samples with approximately 210–400 micrometer particle sizes were taken from each appropriately sifted material. The effect of aging on the particulate materials was investigated by heating the particulates to different temperatures of 800, 1000, and 1200 °C for 6 h. To observe the particulate behavior when exposed to cyclic temperature change, the cyclic heating at 1200 °C was further executed for the particulates for 8 h, and then they were cooled down to room temperature. This process continued until reaching 500 h of heating. The characterization of agglomeration behavior after heating at high temperature, thermophysical and optical properties represented by weighted solar absorptance, and specific heat properties were investigated. An EDX and XRD study relating to an elemental analysis was conducted on the crystalline phases that existed in the samples that were more susceptible to agglomeration. The following are the main conclusions:

1. We observed that all of the particulates showed considerable agglomeration, except for the white sand.
2. The color of the red sand changed entirely to white.
3. The white sand turned more whitish, while the Carbobead CP exhibited a slight color change compared to the original.
4. There was slight agglomeration in the Carbobead CP sample at 1200 °C. According to the appearance, the solar absorptance was very low for the red sand (40.46%) and white sand (36.11%), while the Carbobead CP showed high solar absorptance (85.04%) after aging.
5. The white sand and red sand turned completely into pure silica after the cyclic heating, and this was evident in the heat analysis and qualitative elements.
6. The chemical composition of the white sand was almost constant after the cyclic heating, indicating that the material had high durability and reliability. Furthermore, matching the c_p_ values with the pure silica values after the aging test showed the positive effects of cyclic heating at high temperatures. However, the lower solar absorptance, which was slightly decreased after the cyclic heating, remains as the major issue.
7. EDX and XRD analyses of the red sand showed that the aging test converted its chemical composition entirely into pure silica by removing all the other elements and impurities.
8. A slight decrease in the solar absorbance was observed in the Carbobead CP from what it was “as received” after cyclic heating. This decrease did not continue after 32 h of cyclic heating. The EDX and XRD results showed an almost similar chemical structure stability after cyclic heating.

## Figures and Tables

**Table 1:** Characterization of candidate particulate materials.

Material	White Sand	Red Sand	Ilmenite	Carbobead CP
Average Particle Diameter (µm)	210 to 425	210 to 425	210 to 425	300
Shape	Irregular	Irregular	Irregular	Regular
Cost (per kg)	USD 0.03 to USD 0.05	USD 0.03 to USD 0.05	USD 0.03 to USD 0.07	$1
Optical Properties (As Received)	Low	Low	High	High
Local Availability	Widely available	Widely available	Widely available	Not available
Specific Heat * (kJ/kg°C)	-	-	-	$c_{p}=0{.365\;T}^{0.18}$ 50 °C ≤ *T* ≤ 1100 °C
Chemical Composition *	-	-	-	75% Al_2_O_3_, 11% SiO_2_, 9% Fe_2_O_3_, 3% TiO_2_, and 2% others

* Specific heat and chemical composition of the Carbobead CP was measured at Sandia National Laboratories using a differential scanning calorimetry technique [46].

**Table 2:** Photographic images of the candidate particulate materials after heating at different temperatures.

Material	Fresh	800 °C	1000 °C	1200 °C	Cyclic Heating for 500 h at 1200 °C
White Sand	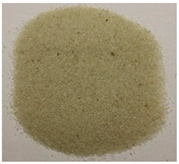	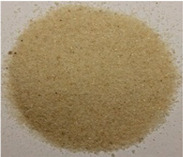	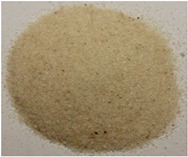	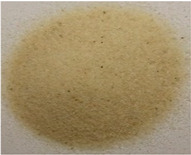	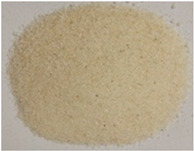
Red Sand	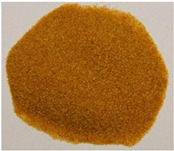	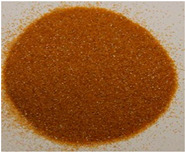	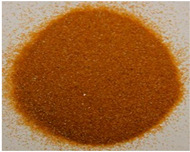	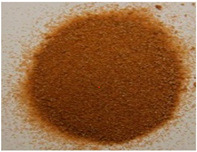	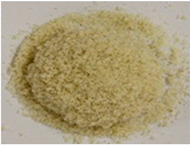
Ilmenite	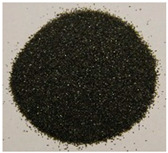	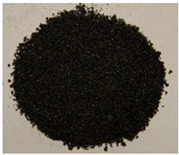	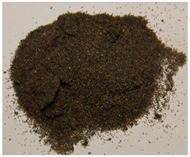	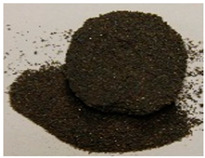	
Carbobead CP	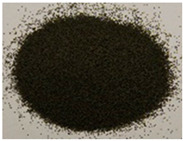			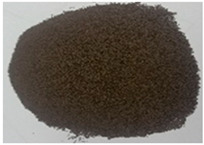	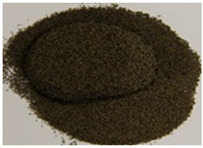

**Table 3:** Solar absorptivity (Abs %) measurements for different particulate materials “as received” and after the aging test.

Sample	Fresh	800 °C	1000 °C	1200 °C (500 h)
White Sand	41.48 ± 1.00	41.29 ± 0.36	37.20 ± 0.62	36.11 ± 0.84
Red Sand	64.57 ± 0.3	62.78 ± 0.34	61.20 ± 0.24	40.47 ± 1.3
Ilmenite	90.94 ± 0.92	85.66 ± 0.81	92.02 ± 0.37	
Carbobead				85.04 ± 0.13
(Abs %) measurements of Carbobead “as received” after heating at 1200 °C				
Carbobead	4 h			83.54 ± 0.21
Carbobead	32 h			85.25 ± 0.12

**Table 4:** Elemental composition of white sand.

Element	White Sand (Weight %)				
	Fresh	800 °C	1000 °C	1200 °C	1200 °C—500 h
O	64.94	66.83	67.40	63.52	57.59
Al	1.16	1.00	1.17	1.26	0.99
Si	33.90	32.17	31.41	35.22	41.42
Total	100.00	100.00	99.99	100.00	99.99

**Table 5:** Elemental composition of red sand.

Element	Red Sand (Weight %)				
	Fresh	800 °C	1000 °C	1200 °C	1200 °C—500 h
O	64.99	60.43	61.17	64.12	59.58
Mg	0.55	0.71	0.41	0.51	-
Al	2.14	2.59	2.26	1.91	1.80
Si	29.54	32.30	30.71	29.96	38.62
K	0.22	0.26	0.48	0.35	-
Ca	0.42	0.71	2.01	0.79	-
Ti	-	-	0.34	-	-
Fe	2.12	3.00	2.65	2.35	-
Total	99.99	100.00	100.03	100.00	100.00

**Table 6:** Elemental composition of CARBOBEAD CP.

Element	CARBOBEAD CP (Weight %)			
	Fresh	1200 °C—4 h	1200 °C—32 h	1200 °C—500 h
O	49.95	50.91	51.37	51.59
Al	31.38	40.25	40.75	39.42
Si	6.76	2.20	3.52	4.79
Fe	11.27	3.80	2.84	4.19
Ti	0.65	1.90	1.52	-
Mn	-	1.43	-	-
Total	100.00	100.43	100.00	99.99

**Table 7:** Bulk density and particle density (kg/m^3^) for particulate materials “as received” and after each thermal treatment.

Material	Bulk Density				Particle Density			
	As Received	800 °C—6 h	1000 °C—6h	1200 °C—500 h	As Received	800 °C—6 h	1000 °C—6 h	1200 °C—500 h
**RS**	1610.1 ± 0.6%	1639.6 ± 1.2%	1590.5 ± 1%	1319.9 ± 2%	2692.8 ± 0.9%	3880.2 ± 11%	3528.9 ± 8.5%	4457.5 ± 13%
**WS**	1549.9 ± 0.7%	1606.8 ± 1%	1710.5 ± 1.1%	1726.5 ± 1.6%	2792.4 ± 1.3%	3023.5 ± 3.5%	3054.7 ± 4.2%	3397.1 ± 8.3%
**Note:** The heat treatment of the Carbobead CP was only done at 1200 °C for different periods.								
**CP**	**As Received**	**4 h**	**32 h**	**500 h**	**As Received**	**4 h**	**32 h**	**500 h**
	2029.0 ± 0.9%	2138.7 ± 1.3%	2197.3 ± 1.5%	2077.4 ± 1.2%	3248.8 ± 1.0%	4193.4 ± 6.6%	4398.0 ± 7.3%	3818.2 ± 8.4%

## Data Availability

Not applicable.
